# Identification of disease causing loci using an array-based genotyping approach on pooled DNA

**DOI:** 10.1186/1471-2164-6-138

**Published:** 2005-09-30

**Authors:** David W Craig, Matthew J Huentelman, Diane Hu-Lince, Victoria L Zismann, Michael C Kruer, Anne M Lee, Erik G Puffenberger, John M Pearson, Dietrich A Stephan

**Affiliations:** 1 Neurogenomics Division, Translational Genomics Research Institute (TGen) Phoenix, Arizona 85004, USA; 2 Clinic for Special Children, Strasburg, PA 17579, USA

## Abstract

**Background:**

Pooling genomic DNA samples within clinical classes of disease followed by genotyping on whole-genome SNP microarrays, allows for rapid and inexpensive genome-wide association studies. Key to the success of these studies is the accuracy of the allelic frequency calculations, the ability to identify false-positives arising from assay variability and the ability to better resolve association signals through analysis of neighbouring SNPs.

**Results:**

We report the accuracy of allelic frequency measurements on pooled genomic DNA samples by comparing these measurements to the known allelic frequencies as determined by individual genotyping. We describe modifications to the calculation of *k*-correction factors from relative allele signal (RAS) values that remove biases and result in more accurate allelic frequency predictions. Our results show that the least accurate SNPs, those most likely to give false-positives in an association study, are identifiable by comparing their frequencies to both those from a known database of individual genotypes and those of the pooled replicates. In a disease with a previously identified genetic mutation, we demonstrate that one can identify the disease locus through the comparison of the predicted allelic frequencies in case and control pools. Furthermore, we demonstrate improved resolution of association signals using the mean of individual test-statistics for consecutive SNPs windowed across the genome. A database of *k*-correction factors for predicting allelic frequencies for each SNP, derived from several thousand individually genotyped samples, is provided. Lastly, a Perl script for calculating RAS values for the Affymetrix platform is provided.

**Conclusion:**

Our results illustrate that pooling of DNA samples is an effective initial strategy to identify a genetic locus. However, it is important to eliminate inaccurate SNPs prior to analysis by comparing them to a database of individually genotyped samples as well as by comparing them to replicates of the pool. Lastly, detection of association signals can be improved by incorporating data from neighbouring SNPs.

## Introduction

The ability to genotype hundreds of thousands of single nucleotide polymorphisms (SNPs) across the genome and to perform association analysis between cases and controls provides, for the first time, a discovery-based approach for determining the underpinnings of complex human genetic disorders. Technologies from Affymetrix (microarray-based GeneChip^® ^Mapping arrays), Illumina (BeadArray™), and Sequenom (MassARRAY™) are now available with sufficient density to detect linkage disequilibrium between informative SNPs and nearby disease-causing nucleotide variants through non-hypothesis based whole-genome association scans for certain populations [1-3].

Several practical issues make whole-genome association studies by utilizing individual genotyping difficult to implement [4]. Power estimates predict that somewhere on the order of a thousand cases and control subjects must be genotyped to detect allelic differences of <5% between the cohorts, as well as to detect rare alleles which may be causative in only a subset of the cohort [5]. Additionally, population stratification and allelic imbalance may identify SNPs that have statistically significant allelic frequency differences yet have no relation to the disease [4,6,7]. Whole-genome association studies are now technologically possible, though the cost is several million dollars if samples are individually genotyped. Here we describe the validation of pooling genomic DNA samples as a rapid pre-screening to detect disease-causing loci for a few thousand dollars on SNP genotyping microarrays.

It is possible to identify SNPs that have significant differences in allelic frequencies between two populations while saving a significant amount in resources by pooling genomic DNA and then SNP genotyping on a single microarray, or preferably on a series of replicated arrays. Indeed, a limited number of studies have been conducted that demonstrate the possibility to predict accurately the allelic frequencies of a SNP from a pooled sample on a microarray, and, in fact identify quantitative trait loci [8-12]. Typically, these studies have validated the pooled allelic frequencies by later individually genotyping between ten to a few hundred SNPs. The most elegant validations of pooling have used indirect approaches, such as spiking a single individual of known genotype into a pooled group with unkown genotypes [9].

One cannot realistically expect all probes on a microarray to function equally, especially considering that the objective of these platforms is to identify allelic differences of 0%, 50%, and 100%. Indeed, as platforms move to 100,000+ SNPs, the ability to select preferentially the best performing SNPs, such as was done in the design of the Affymetrix 10K GeneChip^®^, will likely be compromised. As a result, our prediction is that many SNPs will be unreliable for pooling, and thus may be more likely to lead to false positives. In a pooling study, limiting false positives that are a result of the assay, rather than the underlying population, will be a major factor in being able to realistically identify SNPs that can predict disease status. In this study, we investigated the reliability of SNP allelic frequency measurements as determined from pooling genomic DNA samples on SNP mapping arrays. We further demonstrate our ability to identify poorly predictive SNPs prior to analysis.

## Results

We compared the predicted allelic frequencies from pools of genomic DNA to the known allelic frequencies determined by individual genotyping in order to establish the accuracy of pooling. The goal was to compare allelic frequencies for all the SNPs on a microarray, since not all SNPs will be equally accurate for the prediction of frequencies. Inaccurate SNPs are expected to be problematic as microarrays progress to probe hundreds of thousands of SNPs, whereby SNPs are chosen primarily for their physical position in the genome and not for their reproducibility. Indeed, in order to identify 11,500 SNPs for the Affymetrix 10K GeneChip^® ^Mapping Array nearly 500,000 SNPs were screened by Affymetrix for reliability in the assay [13].

### Individual genotyping of SNPs for 107 samples

Allelic frequencies for 10,205 SNPs on 107 samples were determined by individually genotyping on the 10K GeneChip^®^. These samples were genotyped over a one-year period; therefore, some samples were genotyped on version 1.0 of this platform and others on version 2.0. Only SNPs genotyped on both platforms were utilized for this study. Accuracy of SNP calls was approximately 99.8%, as determined by inheritance errors in family pedigrees, in line with the accuracy reported by Affymetrix (99.9%) [13]. We found no significant decrease in accuracy between the two versions of the 10K GeneChip^®^. The average percentage of SNPs called across all 107 samples was 90.9%. Only individuals with a call rate above 80% were included in the present study. For example, 2,783 SNPs were called for all individuals and 3,525 SNPs were called for >98% of individuals. In our experience using this platform on over 4,000 samples we determined that the call rate is highly dependent on DNA quality and that high quality genomic DNA yields a call rate of 95–98%. The samples used in this study have been collected over several years with variable DNA quality. It is to be expected expect that large-scale whole-genome association studies will also be forced to utilize DNA of less than optimal quality since hundreds to thousands of individuals are needed. Thus the genomic DNA used in this study will likely be representative of what could be expected in a whole-genome association study on a disease where several-thousand individuals are needed.

### Construction of pools

Pools were created in triplicate from the individually genotyped samples. The individuals were from the Old Order Amish and Old Order Mennonite populations of southeastern Pennsylvania [14]. Pool 1 consisted of 52 individuals, Pool 2 consisted of a different 52 individuals, and Pool 3 consisted of 3 patients who died of a form of sudden infant death syndrome known as SIDDT and had a known region of identity-by-descent (shared a pre-defined allele on all six chromosomes across the case cohort) [14]. This region was defined on the 10K microarray by 13 consecutive autozygous SNPs, 6 of which were informative. All DNA was quantitated using PicoGreen reagent (Molecular Probes, Eugene, Oregon) to ensure equal amounts were contributed to the pool from each individual. These three pools were then genotyped in replicates of three on the 10K GeneChip^®^. In all, 9 microarrays were used for the pooled genotyping compared to 107 microarrays for the individual genotyping.

### Calculation of allelic frequencies from pooled samples

The predicted allelic frequencies from pooled genotype samples were calculated for each SNP using a *k*-correction factor based on their derivation from over 3,000 individuals genotyped on the 10K GeneChip^®^. The training set consisted of 3,000 individuals that were genotyped in our lab within the past year. All had call rates above 80% with an average call rate of 95%. None of the 3,000 individuals used for calculation of *k*-correction factors were included in the pooled genomic DNA.

**Figure 1:**
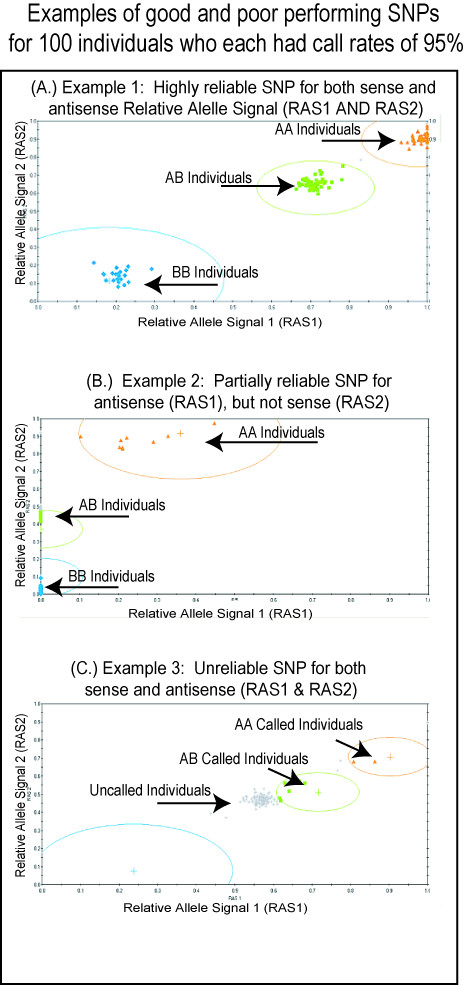
Example of RAS statistics for three SNPs based on genotyping of 100 individuals with an average call rate of all SNPs greater than 98%. These example SNPs illustrate how SNP call reliability can vary both between SNPs and within the same SNP, as measured by RAS1 and RAS2 values. Blue spheres are BB individuals, orange triangles are AA individuals, and green squares are AB individuals, grey stars are "Not Called".

The purpose of the *k*-correction factor is to allow for calculation of a predicted allelic frequency from peak heights, or in this case fluorescence signal, whereby *p *= *A*/(*A*+*kB*) [15]. *K*-correction factors have recently become well established and have been used successfully in primer extension assays whereby measurements in SNP allelic frequencies on pooled genomic DNA have been taken by HPLC, mass spec, and by fluorescence in TAQMAN assays [15-19]. For the 10K Mapping array assay, *p *is the predicted allelic frequency of the A allele, *A *is the fluorescent signal intensity measure of the A allele, and *B *is the fluorescent signal intensity measure of the B allele. The *k*-correction factor can be calculated for a given SNP using a heterozygote who is AB, effectively 50% A and 50% B. Conveniently, output of the Affymetrix GeneChip software for the Affymetrix 10K Mapping Array includes Relative Allele Signal (RAS) values which have been previously used to determine *k*-correction values (see Figure 1) [11]. Generally, RAS = A/(A+B). Here, A refers to the median match/mismatched differences of the major allele and B for the minor allele (Affymetrix Technical Manual). There are two RAS values, RAS1 (sense) and RAS2 (antisense) since both sense and antisense directions are probed.

Whereas *k*-correction factors based on the Affymetrix 10K GeneChip^® ^Mapping Array have been previously calculated directly using only heterozygous RAS values [11], we suggest that this can be improved upon since the RAS values are generally not 0 or 1 for homozygotes (See Figure 1). Indeed, we observed significant deviation for many SNPs, which could potentially add significant bias (see discussion) [11]. Thus for each SNP, we normalized RAS values, referred to as nRAS, using the individuals from the training set that were AA (normalized to 1) and BB (normalized to zero). Without this normalization, predicted frequencies will be systematically biased as the pooled samples approach homozygosity. Thus nRAS_x _= (RAS_x_-AA_ave_)/(BB_ave_) where AA_ave _is the average RAS_x _score for individuals AA in the training set, and BB_ave _is the RAS_x _score for individuals BB in the training set. The value of X refers to whether the calculation is for RAS1 or RAS2, and nRAS values are calculated for both RAS1 and RAS2. Thus, two predictions of allelic frequency are obtained: one from RAS1 and one from RAS2. Each RAS variable has distinct variability, and as shown in Figure 1(b), RAS1 may be very precise with low variance, while RAS2 may exhibit high variance, and vice versa. Averaging the two RAS values will mask the RAS value with lower variance. Because of this independent variability, we do not recommend averaging RAS1 and RAS2 for all SNPs as was suggested in other pooling studies [8,10,12]. Rather, we recommend treating the two RAS values as separate experiments, and preferably removing RAS values with the greatest variance prior to analysis.

Values making up each of the RAS1 and RAS2 mean values are provided for homozygous AA, homozygous BB, and heterozygous individuals on a website based on our 3,000 person database which is being made available to the public as part of this publication. These *k-correction *factors derived from RAS1 and RAS2 values using this training dataset are available at [20] and as supplementary material.

### Comparison of allelic frequencies: Pooling vs. individual genotyping

**Figure 2:**
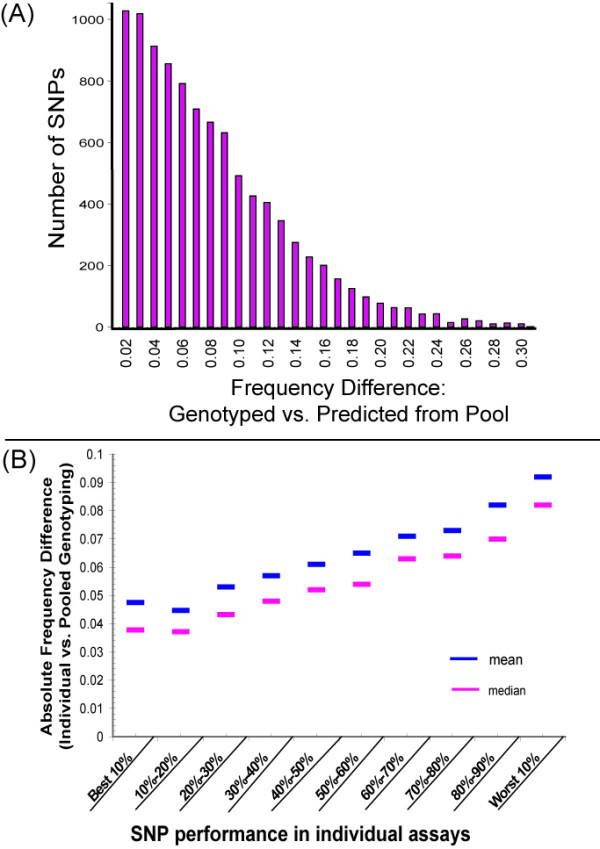
(A) Allele frequency differences between individual and pooled genotypes. Histogram representing the total number of SNPs at each allele frequency difference between individual and pooled samples. (B) Accuracy of predicted SNP frequencies increases for those SNPs that perform well on Mapping 10K individual assays and decreases for poorly performing SNPs. The mean and median absolute difference between the predicted allelic frequency and individually genotyped allelic frequencies are shown vs. the binned performance of SNPs on individual assays. Performance is ranked by the frequency of calls in a set of 3,000 individually genotyped samples.

For the 10,205 SNPs on the Affymetrix 10K GeneChip^® ^we found a median difference in allelic frequency between individually genotyped samples and pooled samples of 5.1%, a mean difference of 6.3%, and a standard deviation of 4.9%. Figure 2 shows a histogram for all the SNPs and their difference between the predicted frequency from the pools and the individual genotypes data. Other studies have reported slightly lower differences between pooled and individually genotyped methods for determining allelic frequencies (3–5%) [8,9,12]. Many reasons are likely for this difference: We used DNA that was collected over ten years and was of varying quality; we also compared all the SNPs on the microarray rather than selecting only a few SNPs for comparison. Realistically, the greater difference seen in this study may be more representative of large association studies, in which thousands of genomic samples of varying quality are pooled.

### Identification of assay false positives

While the differences in frequencies between pooled and individual genotyped samples show that calculating frequencies from pooled samples is highly accurate, it is perhaps of greater importance that we are able to predict those SNPs that are unreliable and largely inaccurate. Assays genotyping 500,000 SNPs will likely not have the ability to be as selective and thus are likely to provide a large number of SNPs that do not reliably quantitate allelic frequencies from pooled genomic samples. As shown in Table 1, we found that the 100 SNPs most likely to give a "NoCall" in individual genotyped samples more often gave unreliable predictions of allelic frequencies in pooled samples. Furthermore, as shown in Figure 2b, those SNPs that are the worst 10% (in terms of % called for individual genotypes) also gave rise to higher allelic frequency differences.

**Table 1:** Inaccurate SNPs with the largest difference between SNP allele frequencies when genotyped individually vs. calculated from pooled DNA can be partially predicted. Nearly 40% of the SNPs found to be the 100 most inaccurate SNPs were also either (a) one the 500 worst performing SNPs in individual genotyping or (b) had the largest variability between replicates in the pool.

**All SNPs**	**(a) 500 worst performing SNPs Criteria: NoCalls on 3000 person database**	**(b) 500 worst performing SNPs Criteria: Pool variability from 3 replicates**	**SNPs found in (a) or (b)**	**Inaccuracy (predicted vs. genotyped)**
**100 most inaccurate SNPs (individual genotyped vs. pooled)**	24.2%	27.3%	38.6%	27.2%
**500 most inaccurate SNPs (individual genotyped vs. pooled)**	12.5%	14.5%	22.1%	20.2%
**Remaining 9605 SNPs**	4.4%	5.5%	8.6%	5.0%

**Figure 3:**
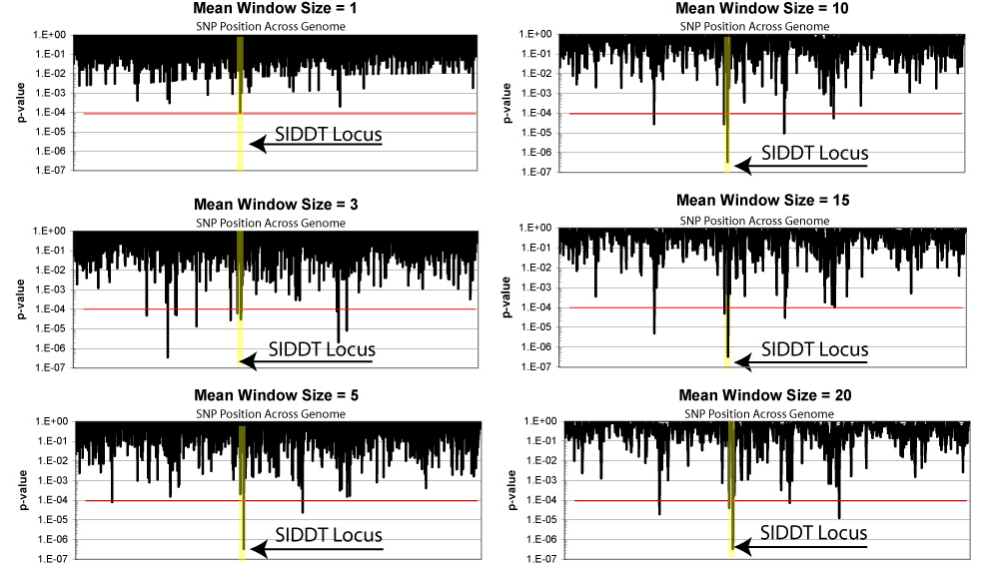
Identification of the SIDDT locus from pooled genomic DNA by calculating the mean test-statistic for a rolling window of consecutive SNPs. The moving window was determined across the genome and the p-value was calculated from a distribution of 400 bootstraps of the original dataset. Mean window sizes of 1, 3, 5, 10, 15, and 20 are shown and the SIDDT locus is highlighted in yellow. The SIDDT disease locus is the top region for window sizes of 1, 5, 10, 15, and 20.

We found that rarely called SNPs are also likely to be called inaccurately (Table 1 and Figure 3c). In this case, constructing *k*-correction factors and predicting allelic frequencies will be unreliable for these SNPs, even if pooled replicates show low variability. SNPs with the highest variance in pool replicates were also unreliable. As a practical measure, we found that applying both filters for too many "NoCalls" in a training set and having a high variance in pooled replicates was more effective than either measure alone. We could identify 1/3^rd ^of the worst performing SNPs (greater than 12% difference), by removing the worst performing 5% of SNPs based on variance in pool replicates and removing the worst performing 5% of SNPs based on excessive "No Calls" when individually genotyped. Consequentially, the removal of those SNPs that are either poorly called in a training set of individually genotyped samples or highly variable across pooled replicates significantly decreases the number of false positives. The number of SNPs removed should maintain a balance between retaining dense SNP coverage and excluding those SNPs more likely to give false positives. Ultimately, removing potential false positives will be a compromise between the coverage of the SNP microarray and the genetic diversity of the population.

It is of interest to note that allelic frequencies calculations were more accurate as SNPs approached homozygosity. For example, for those SNPs with allelic frequencies from 0% to 20% and from 80% to 100% the mean difference was 5.2% vs. a mean difference of 7.1% for SNPs with allelic frequencies between 20% and 80%. This finding may be due to inaccuracies in the assays as SNPs approach 50%, since the variance for heterozygotes is higher than the variance measured for homozygotes.

### Identification of a disease locus from genotyping of pooled samples

In order to assess whether it is feasible to use pooled genotyping to identify the genetic locus for a disease, we created case and control pools for the disease sudden infant death with dysgenesis of the testes (SIDDT) and a pool of Amish control individuals.

A test for proportions was employed to detect statistical differences between cases and controls. This test-statistic is more often used in pooled studies since frequency data are generally not whole-integers [19]. Shown in equation 1 is the calculation for the test statistic (T) where *f*_*case *_is the allele frequency for the case group and *f*_*control *_is the allele frequency for the control group.



The distribution follows an approximate χ^2 ^distribution with one degree of freedom. The SNP with the highest significance, rs949748, had a p-value of 0.00016 and was in the SIDDT locus at chromosome 6q21. However, it is expected that the SNP with the lowest p-value will not always be at the correct disease locus. Even strong single SNP association signals will likely be obscured in the noise when 500,000+ SNPs are probed. Thus we employed a moving window whereby the mean test-statistic of several consecutive SNPs was calculated at each SNP position across the genome. The objective of the moving window was to leverage the fact that neighbouring SNPs will likely be in linkage disequilibrium, whereby one SNP is at least partially predictive of the neighbouring SNP. The number of SNPs contained in the moving window was varied between 1 and 25. Shown in table 2 is the rank of the 6q21 region for varying window sizes. It is of interest to assess sensitivity of this windowing approach to SNPs within the region. Thus, we consecutively removed the top three SNPs contributing to the overall association signal. Removing the first two SNPs has little effect on detecting the association signal. The 6q21 region remains the most significant for window sizes of four and greater even when these top two SNPs are removed. In comparison, all three top SNPs has a marginal effect, lowering the rank of the region from highest to within the top ten.

**Table 2:** Identification of disease locus using a moving window. SNPs were ranked by test statistics and sorted by physical position. The average was calculated for a moving window of consecutive SNPs across the genome. The region 6q22.1 was already known to contain the mutation leading to the SIDDT. The rank of region 6q22.1 for a various window sizes in shown in the second column. In the 3rd, 4th, and 5th columns, the top 1, 2, and 3 SNPs were removed from the 6q22.1 regions to probe sensitivity of window size.

**# SNPs Averaged in Moving Window**	**6q22.1 Rank Region (All SNPs)**	**6q22.1 Region Rank (Exclude Top 1 SNP in Region)**	**6q22.1 Region Rank (Exclude Top 2 SNPs in Region)**	**6q22.1 Region Rank (Exclude Top 3 SNPs in Region)**
1	1	22	24	60
2	11	11	19	11
3	6	6	6	14
4	1	1	1	2
5	1	1	1	8
6	2	2	2	3
7	1	1	1	13
8	1	1	1	3
9	1	1	1	9
10	1	1	1	3

To compute the statistical significance of averaged test-statistics, we used a permutation test. With this approach the consecutive order of SNPs was randomized in four hundred separate bootstrapped datasets. P-value statistics were calculated from the distribution of these datasets. Shown in Table 2, the SIDDT locus (6q22.1-q22.31) was generally revealed as the most significant region of association for window sizes between 4 and 20 SNPs.

It will not always be the case that SNPs are in linkage disequilibrium and a windowing-based approach will be effective. The permutation statistics can be used to test this scenario in order to see if the frequency of a given mean window test-statistic is indeed significant. The Old Order Amish and Mennonite populations used in this study arise from a population founded in approximately the sixteenth century with expectedly larger regions of identity by descent. The Amish and Mennonites are not one large isolated population. It is more accurate to say that both these populations derive from the Swiss Anabaptists (circa 1525). These groups are socially and genetically unique even though both came from the same geographical region. Thus undoubtedly some stratification exists between our two cohorts and it is encouraging that the correct region was easily identifiable despite any stratification [21].

Based on previous research in this population, the 10K Mapping Array was anticipated to be of sufficient density whereby many of the SNPs would be in relative linkage disequilibrium throughout this regional population. Indeed, the permutation statistics of moving windows support this notion as the 6q21 region shows a p-value of <1e-6 for window sizes of 10 SNPs and greater, far lower than would be expected with ~10,000 SNP measurements. Other methods have been developed that reduce noise using haplotype data from SNPs in linkage disequilibrium [22]. In the absence of this haplotype data, which may often be the case, it is encouraging that the very straightforward statistical approach described here is effective at identifying the correct locus.

## Discussion

Our results show that (1) pooling genomic samples is highly accurate; (2) unreliable SNPs most likely to give false-positives can be largely identified and removed prior to association analysis; and (3) a moving window of averaged test-statistics can be used to detect association signals. Additionally, we have described modifications as to how allelic frequencies are calculated from RAS values of pooled samples that remove systematic biases.

Pioneering work on pooling studies by other research groups has shown that the average Relative Allele Signal (RAS_ave_) can be effectively used to derive *k*-correction factors by *k *= RAS_AVE_/(1-(RAS_AVE_), and as such, can be used to accurately predict allelic frequencies [8,11,12]. Pooling studies are intended to be screening approaches. RAS values are highly convenient since they are generated by the Affymetrix GDAS software on the 10K platform and fairly intuitive to understand. We suggest significant improvements to this innovative approach that will remove biases; allow for continued use of RAS values; and result in more accurate predictions. These improvements focus on lowering the number of false positives due to added variance or systematic biases, since the utility of pooling-based approaches will be based on how one can detect association signals given a high number of false positives.

First, RAS1 and RAS2 should not be averaged since they are separate probe sets with distinct variances. One may unnecessarily propagate unwanted variance by averaging. For example in Figure 1b, it is clearly visible that RAS2 is highly predictable of the particular SNP allele whereas RAS1 is highly inaccurate. In this case, averaging RAS2 and RAS1 will produce a RAS_AVE _value that is less accurate than RAS2 alone. We suggest instead that these values be treated as separate measures, each with their distinct variance. In the case of RAS values with a large variance, these values should not be used due to the increased chance of a false positive.

Second, we highly recommend that RAS values for each SNP be normalized prior to calculation of allelic frequencies. When these values are not normalized prior to calculating a predicted allelic frequency a significant bias is introduced since the RAS values, as produced by the Affymetrix GDAS software, generally are not 0.0 or 1.0 for homozygous BB and AA respective alleles. Indeed, on a training set of 1000 individuals we found that 34% of SNPs who were called AA had a RAS value less than 0.9 and 35% of SNPs called BB had a RAS value greater than 0.1. This bias can be seen in an example calculation using *k*-correction factors derived from a typical RAS value directly obtained from the GDAS software. For example, the average RAS1 for a given SNP of an AA individual may be 0.9, the average RAS1 for a heterozygous individual may be 0.5, and the average RAS2 for a BB individual may be 0.1. When one uses the approach outlined by Butcher, *et al*, the *k*-correction factor is 1.0, whereby the RAS value of the average heterozygote is divided by one minus this value [10]. In a pooled sample, the same SNP is expected to have a RAS value of 0.9 if it is completely homozygous for AA. However, using the *k-correction *approach on non-normalized RAS values, one would predict an allelic frequency of 90%, whereas the actual frequency is 100%, a bias of 10%. These biases would be most pronounced as pools approach dominance by one allele type, as would often be the case for a SNP highly associated to a disease.

While RAS values are readily obtainable from the Affymetrix software for the 10K GeneChip^® ^arrays, they are not provided for the 100K or 500K. This is partly due to the fact that RAS values are no longer used to make a SNP call. We have developed a simple Perl script which generates RAS values, still useful in pooling, for the 100K and 500K Affymetrix GeneChip^® ^platform from CHP files. This tool is available on our website [23]. While one may use these RAS values to find obvious differences in cases and controls, for many SNPs allelic frequencies are not linearly dependent on the RAS values; thus, one should calculate allelic frequencies when possible to reduce uneven biases between different SNPs.

Additionally, we are making public on the same site both normalized and non-normalized *k-correction *factors derived from over 3,000 genotyped individuals for the 10K version 2.0 SNP genotyping platform. Other research groups have created central repositories for *k-corrections *using non-normalized RAS values and we will work with these teams to contribute these values to this valuable centralized resource [11].

## Conclusion

Prior to the investment of large resources into individual genotyping thousands of individuals, one may first consider pooling samples at a low cost to rapidly ascertain gross population stratification concerns and potentially identify the regions of the genome with the strongest association to the trait. The sheer number of SNPs interrogated will lead to a high number of false positives, due to both actual variation in genotype frequencies of the underlying groups and to technical variance. We demonstrate that technical variance can be detected *a priori *for each SNP using training sets from large numbers of individual microarrays or by replicates of pooled samples. We further show that despite the issues of population stratification, admixture, and subgroups that are difficult to detect when pooling, the cost savings make pooling a first step that we suggest should logically precede the investment of millions of dollars. We describe here a method by which 100K and 500K Affymetrix SNP array data can be parsed into RAS scores and pooled inbalances accurately assessed in an outbred population.

## Methods

### 10K GeneChip^® ^Mapping Array Genotyping

10K SNP genotyping was performed as detailed by Affymetrix on the 10K GeneChip^® ^Mapping 1.0 and 2.0 Arrays [5]. In short, 250 ng of genomic DNA was digested with 10 units of Xba I (New England Biolabs, Beverly, MA) for 2 hours at 37°C. Adaptor Xba (P/N 900410, Affymetrix, Santa Clara, CA) was then ligated onto the digested ends with T4 DNA Ligase for 2 hours at 16°C. After dilution with water, samples were subjected to PCR using primers specific to the adaptor sequence (P/N 900409, Affymetrix) with the following amplification parameters: 95°C for 3 minutes initial denaturation, 95°C 20 seconds, 59°C 15 seconds, 72°C 15 seconds for a total of 35 cycles, followed by 72°C for 7 minutes final extension. PCR products were then purified and fragmented using 0.24 units of DNase I at 37°C for 30 minutes. The fragmented DNA was then end-labeled with biotin using 100 units of terminal deoxynucleotidyl transferase at 37°C for 2 hours. Labeled DNA was then hybridized onto the 10K Mapping Array at 48°C for 16–18 hours at 60 rpm. The hybridized array was washed, stained, and scanned according to the manufacturer's instructions.

The chp_2_ras.pl script processes one or more CHP text files from Affymetrix 10K and 100K SNP chips, calculates RAS1 and RAS2 scores and outputs them in an Excel spreadsheet. Testing shows that for 10K chips, chp_2_ras.pl produces the same scores as those produced by Affymetrix' GDAS software. chp_2_ras.pl is distributed as part of TGen-Array, a collection of Perl scripts and modules that provide parsing and object-oriented interfaces to common microarray files. The script can be downloaded at the TGen bioinformatics website [23].

## Authors' contributions

DWC and MJH performed SNP genotyping, participated in the concept of the paper, and drafted the manuscript. DHL, VLZ, MJH, and AML conducted pooling and SNP genotyping. DWC and JMP performed statistical analysis of the SNP data. DAS participated in study design, coordination, and manuscript drafting. All of the authors have read and approved the final manuscript.

## Supplementary Material

Additional file 1 Calculated k-correction factors for pooling on Affymetrix 10K GeneChip Mapping Array based on 3,000 person database. Click here for file

Additional file 2 The chp_2_ras.pl script processes one or more CHP text files from Affymetrix 10K, 100K, and 500K EA SNP chips, calculates RAS1 and RAS2 scores and outputs them in an Excel spreadsheet. Testing shows that for 10K chips, chp_2_ras.pl produces the same scores as those produced by Affymetrix' GDAS software. GDAS does not calculate RAS values for 100K chips. It should be noted that SNPs on 100K chips do not necessarily contain even numbers of sense and antisense probes and in fact only about 40% have 5 sense and 5 antisense probes. The remaining SNPs have a 6–4 or 7–3 probe bias towards either sense or antisense. This is important because part of the RAS calculation involves taking the median of the "successful" probes and median may not be the best approach if only 3 probes exist in one direction and some may have failed and been discarded. chp_2_ras.pl is distributed as part of TGen-Array, a collection of Perl scripts and modules that provide parsing and object-oriented interfaces to common microarray files. The TGen-Array site contains online documentation for all modules and scripts in the distribution including pages that show the source code so the code and algorithms may be inspected. Click here for file
